# Retromer subunit, CfVps35 is required for growth development and pathogenicity of *Colletotrichum fructicola*

**DOI:** 10.1186/s12863-022-01084-4

**Published:** 2022-08-28

**Authors:** Xi-Ya Li, Sheng-Pei Zhang, Li He

**Affiliations:** 1 Key Laboratory of National Forestry and Grassland Administration for Control of Diseases and Pests of South Plantation, Central South University of Forestry and Technology, Changsha, China; 2 Key Laboratory for Non-wood Forest Cultivation and Conservation of Ministry of Education, Central South University of Forestry and Technology, Changsha, China; 3 Hunan Provincial Key Laboratory for Control of Forest Diseases and Pests, Central South University of Forestry and Technology, Changsha, China

**Keywords:** *Camellia oleifera*, *Colletotrichum fructicola*, CfVps35, Pathogenicity

## Abstract

**Background:**

Tea oil is widely used as edible oil in China, which extracted from the seeds of *Camellia oleifera*. In China, the national oil-tea camellia planting area reached 4.533 million hectares, the output of oil-tea camellia seed oil was 627 000 tons, and the total output value reached 18.3 billion dollars. Anthracnose is the common disease of *Ca. oleifera*, which affected the production and brought huge economic losses. *Colletotrichum fructicola* is the dominant pathogen causing anthracnose in *Ca. oleifera.* The retromer complex participates in the intracellular retrograde transport of cargos from the endosome to the trans-Golgi network in eukaryotes. Vacuolar protein sorting 35 is a core part of the retromer complex. This study aimed to investigate the role of CfVps35 in *C. fructicola*.

**Results:**

The *CfVPS35* gene was deleted, resulting in reduced mycelial growth, conidiation, and response to cell wall stresses. Further analysis revealed that CfVps35 was required for *C. fructicola* virulence on tea oil leaves. In addition, the Δ*Cfvps35* mutant was defective in glycogen metabolism and turgor during appressorium development.

**Conclusion:**

This study illustrated that the crucial functions of CfVps35 in growth, development, and pathogenicity.

**Supplementary Information:**

The online version contains supplementary material available at 10.1186/s12863-022-01084-4.

## Background

*Camellia oleifera* is native to China and it is mainly used to produce edible oil [1]. It is widely distributed in the south Yangtze River area [2, 3]. It provides ecological, social, and economic benefits. The tea oil industry plays an important role in the regional economy of China and has become the leading industry for rural revitalization and targeted poverty alleviation.

Anthracnose is a common and economically important disease in *Ca. oleifera*, seriously affecting the tea oil industry [4]. And this disease can cause premature rot and abscission of oil tea leaves and fruits [5]. Anthracnose of *Ca. oleifera* occurs from early April to late October, peaking in August [6]. *C. fructicola* is a major pathogen causing anthracnose on *Ca. Oleifera* [1].

The retromer complex consists of multiple vacuolar protein sorting proteins, which are associated with the cytoplasmic surface of endosomes and mediate the retrograde transport of transmembrane cargo endosome-to-Golgi transport [7, 8]. The retromer complex allows receptor proteins to be recycled repeatedly, thus avoiding lysosomal degradation and maintaining the metabolic balance [9].

Vps35 is the core protein of the retromer complex. The trimers of VPS35, VPS29, and VPS26 proteins are cargo-selective subcomplexes, which are mainly responsible for the recognition and transport of different receptor proteins [10, 11]. In yeast, Vps35 provides cargo specificity and is directly related to cargo proteins [12, 13]. As a component of the retromer complex, the role of Vps35 in *C. fructicola* is unclear. This study was novel in characterizing the function of CfVps35 in forest fungal pathogens.

## Results

### Identification and phylogenetic analysis of CfVps35

**Fig. 1:**
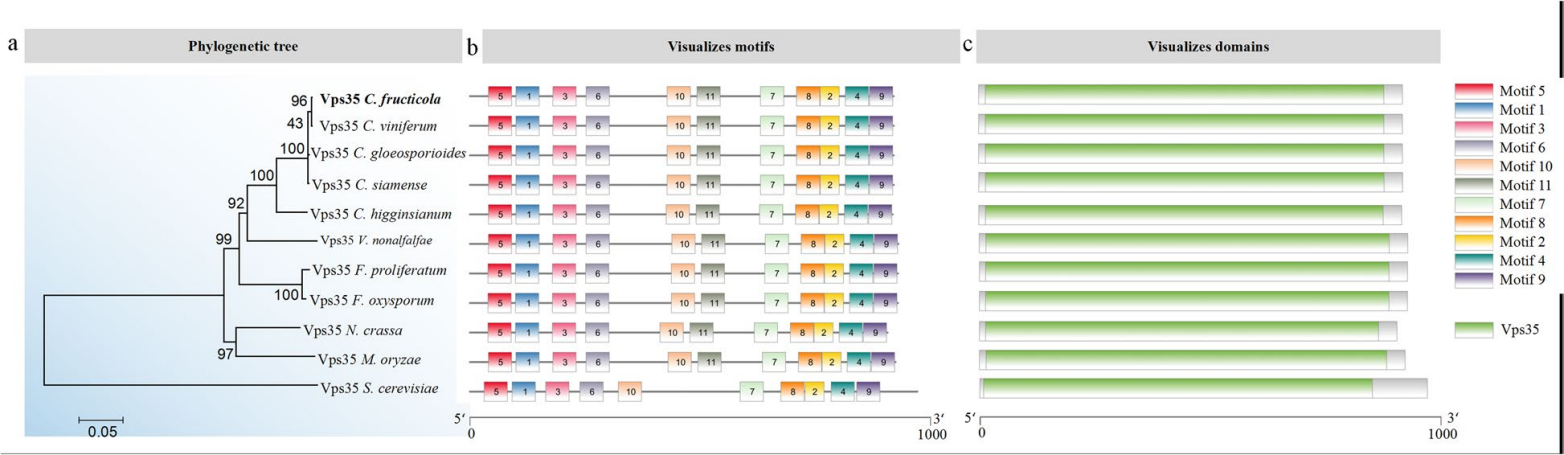
Phylogenetic analysis and Domain prediction of CfVps35. **A** The phylogenetic tree was constructed using the neighbor-joining (NJ) method with 1 000 bootstrap replicates using Mega 7. 0. The number on the branch is the % bootstrap support value; the scale bar indicates the branch length. The GenBank accession numbers are shown as follows: *C. gloeosporioides* (KAF3805764.1); *C. viniferum* (KAF4926567.1); *C. siamense* (XP 036493245.1); *C. higginsianum* (TID04129.1); *Neurospora crassa* (XP 962546.1); *Fusarium proliferatum* (XP 031087386.1); *Fusarium oxysporum* (KAH7216823.1); *Verticillium nonalfalfae* (XP 028497315.1); *S. cerevisiae* (NP 012381.1); *Magnaporthe oryzae* (XP 003712611.1); *Colletotrichum fructicola* (XP_031881347.1). **b** The protein motifs arrangements of CfVps35 and its homologues. Different colors represent different motifs. Straight lines represent total protein length. **c** The protein domain arrangements of CfVps35 and its homologues are visualized in the schematic at the right. Straight lines represent total protein length

We identified a protein homologous to Vps35 of *Saccharomyces cerevisiae* and named it CfVps35. The full length of *CfVPS35* gene was 2900 bp, which encoded 894 amino acids. The domain prediction shows that CfVps35 contains a Vps35 domain composed of 842 amino acids. We also collected CfVps35 homolog sequences in other fungi using BLASTP analysis. Phylogenetic analysis revealed a broad distribution of CfVps35 orthologues from nonpathogenic to pathogenic fungi. CfVps35 shows higher homology with that of *C. viniferum* (99.89% identity and 100% similarity) and lesser homology with *S. cerevisiae* (57.53% identity and 80% similarity) (Fig. 1a)*.* Protein sequence alignment analysis revealed high conservation of nine motifs in the Vps35 domain (Fig. 1b and c).

### Targeted deletion of *CfVPS35*

The coding region of *CfVPS35* was replaced with the *HPH* gene according to the homologous recombination principle to characterize the functions of CfVps35 (Fig. S1a). When the primers *CfVPS35*-5F and H855R were used, amplification of a single band was obtained, while using primers *CfVPS35*-7F/8R did not show any amplification, indicating gene deletion in the mutant (Fig. S1b). The bleomycin-resistant transformants were selected and confirmed using fluorescence and PCR. Then, the complemented strain Δ*Cfvps35/CfVPS35* was obtained and restored all the mutant defects.

### CfVps35 is involved in vegetative growth

**Fig. 2:**
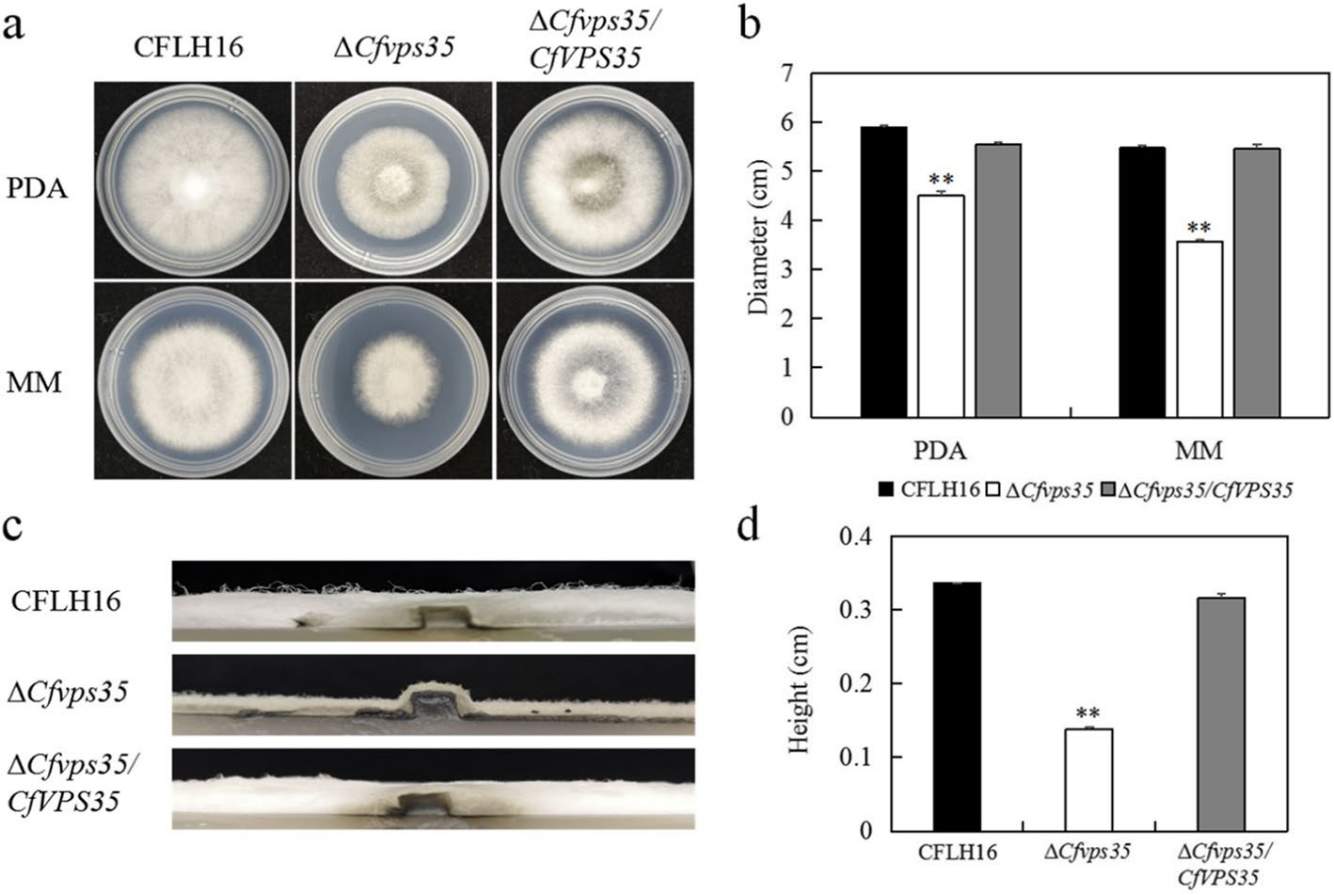
Cfvps35 is involved in vegetative growth. **a** It shows the growth of the ∆*Cfvps35* mutant and the WT and complemented strain, inoculated in PDA and MM media. **b** Statistical analysis of the variations in the growth of the diameter of the mycelium. **c** Side views of mycelial growth are shown. **d** Statistical analysis of the height of aerial hyphae. Error bars are standard deviation, and asterisks represent significance at *P* <0.01 (^**^)

To test growth, WT strain, the ∆*Cfvps35* mutant and the complemented ∆*Cfvps35/CfVPS35* were inoculated onto PDA and MM plates. The ∆*Cfvps35* mutant showed significantly reduced diameter of mycelial growth compared to WT and complemented strains (Fig. 2a and b). Loss of the *CfVPS35* gene affected hyphal growth; the height of the aerial hyphae of the mutant was lower than that of the WT and complemented strains (Fig. 2c and d).

### CfVps35 is involved in sporulation and appressorium formation

**Fig. 3:**
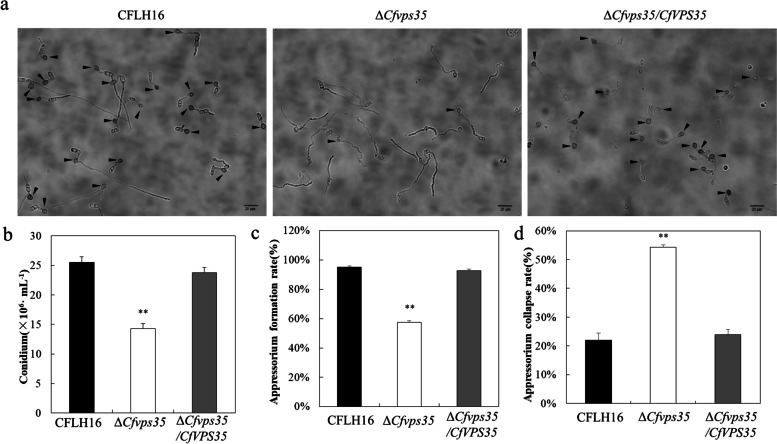
*CfVPS35* gene is involved in sporulation and appressorium formation. **a** Appressoria formation in the WT strain, the ∆*Cfvps35* mutant, and the complemented strain. **b** Conidial quantitative statistical diagram. **c** Statistical analysis of the percentage of appressorium formation. **d** Statistical analysis of the percentage of collapse of the appressoria. Error bars are standard deviation, and asterisks represent significance at *P*<0.01 (^**^)

Conidia play an important role in the infection of *C. fructicola.* The strains were inoculated into the PDB medium to induce sporulation. We found that the spore morphology of the ∆*Cfvps35* mutant was similar to the WT and complemented strain, however, sporulation in the ∆*Cfvps35* mutant was significantly reduced (Fig. 3a and b)

Appressorium is the key infection structure of the fungal pathogen inside the plant tissues. The conidial suspension of *C. fructicola* strains was placed onto the hydrophobic coverslips for 24h to evaluate the appressorium formation of the ∆*Cfvps35* mutant. We observed that the appressorium formation of the ∆*Cfvps35* mutant was reduced (Fig. 3a). The WT strain and the complemented ∆*Cfvps35/CfVPS35* showed the appressoria formation rate of more than 90%, while the mutant strain showed only about 57% (Fig. 3c). We tested the turgor of the appressorium of the ∆*Cfvps35* mutant with a collapse experiment using 2 mol/L glycerin. The collapse rate of the appressorium of the ∆*Cfvps35* mutant was significantly higher than that of the WT and complemented strain (Fig. 3d).

### CfVps35 is involved in penetrability and pathogenicity

**Fig. 4:**
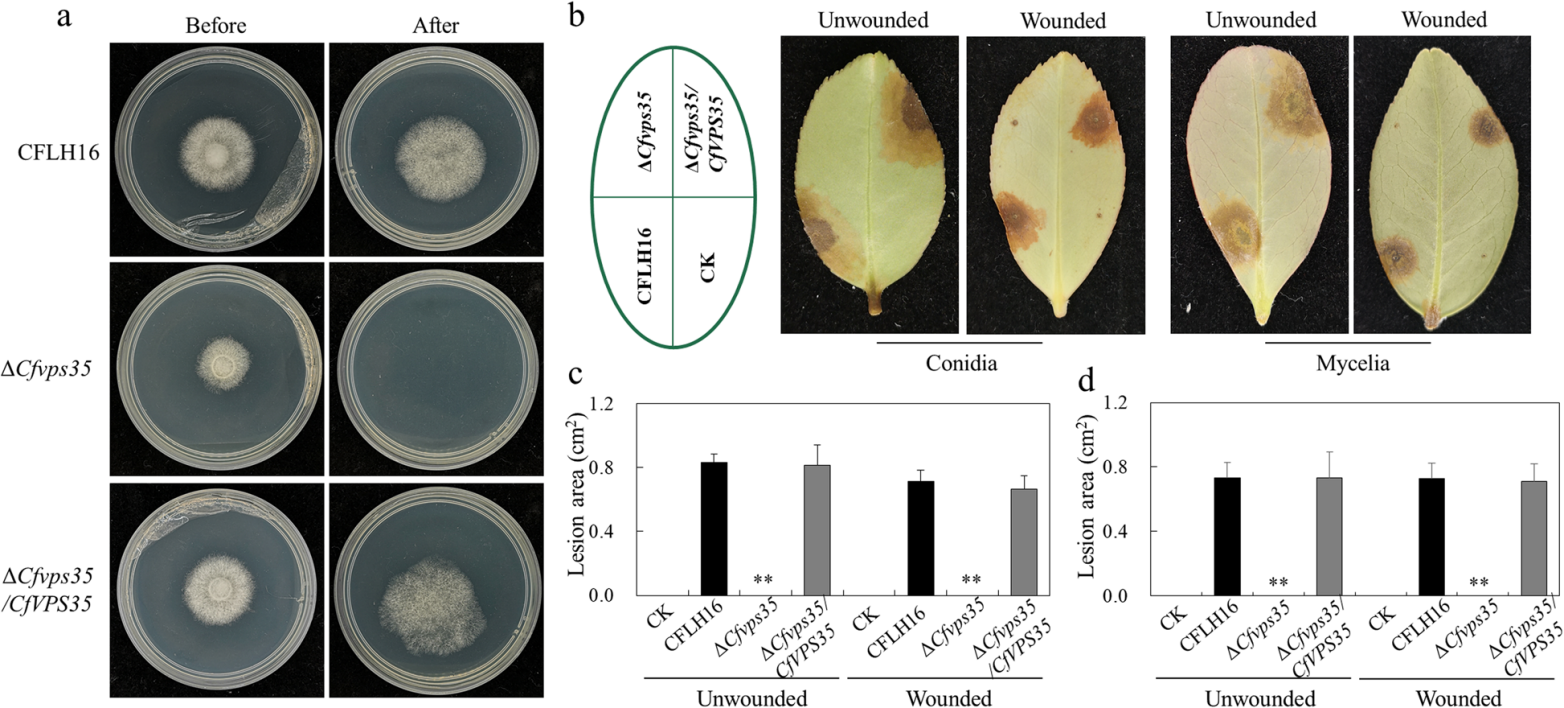
CfVps35 is involved in penetrability and pathogenicity. **a** In the left column (Before), growth of strain CFLH16, mutant ∆*Cfvps35*, and complemented strain Δ*Cfvps35/CfVPS35* on cellophane membranes. In the right column (After), fungal growth after removing the cellophane from the medium. **b** Pathogenicity test strain CFLH16, ∆*Cfvps35* and Δ*Cfvps35/CfVPS35* in unwounded and wounded oil-tea leaves, CK indicates negative control. **c** Statistical analysis of the disease lesion areas inoculated with conidia. **d** Statistical analysis of the disease lesion areas inoculated with mycelia. Error bars are standard deviation and asterisks represent significance at *P*<0.01 (**)

Due to the decrease of appressorium turgor pressure of mutant, we further tested the penetrating ability of each strain to investigate whether the *CfVPS35* gene regulated the penetrability of *C. fructicola*. We found the ∆*Cfvps35* mutant did not pierce the cellophane, and the WT and complemented strain could pierce the cellophane (Fig. 4a).

Additionally, it was determined if the loss of penetrability had an effect on pathogenicity. Unwounded and wounded tea oil leaves were inoculated with conidial suspensions or mycelial blocks of the WT, ∆*Cfvps35* mutant, and complemented strain Δ*Cfvps35/CfVPS35*. After 3 days, the ∆*Cfvps35* mutant showed no lesion in wounded leaves, whereas the WT and complemented strains had typical necrotic lesions (Fig. 4b). After 5 days, the same results were observed on unwounded leaves (Fig. 4b). Statistical analysis of the lesion areas also showed the apparent difference (Fig. 4c and d).

### CfVps35 is involved in glycogen mobilization

**Fig. 5:**
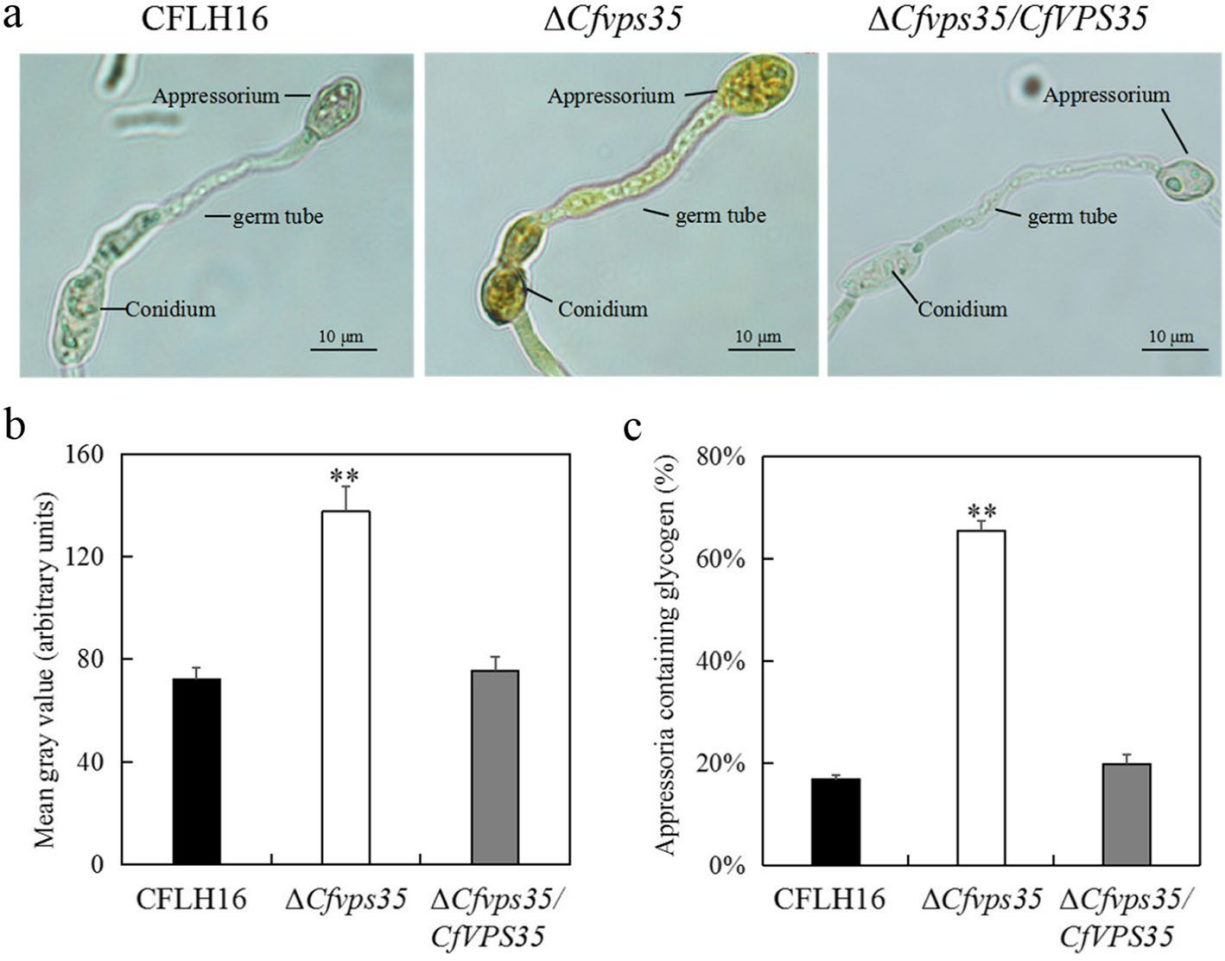
Glycogen translocation of ∆*Cfvps35* mutant during appressoria formation. **a** Glycogen translocation from conidial germination to appressorial formation in the strains. Scale bar =10 μm. **b** Statistical analysis of mean gray value. **c** Statistical of appressoria containing glycogen during appressoria formation. Error bars are standard deviation and asterisks represent significance at *P*<0.01 (**)

Effective transfer of glycogen is required for the appressorium formation, which develops enormous turgor pressure to achieve host penetration [14]. Therefore, we investigated the cellular distribution of glycogen during appressorium development (Fig. 5a). Quantitative analysis of staining intensity showed that the mean gray value of mutants was significantly higher than that of wild-type and complementary strains (Fig. 5b). Staining results showed that the rate of glycogen degradation in the appressorium formation of ∆*Cfvps35* mutant was significantly slower. In the WT and complemented strain, 80 % of the appressorium glycogen were almost degraded at 24 h, while in the ∆*Cfvps35* mutant, 60 % of the spores and appressorium glycogen were still present at 24 h (Fig. 5c). Therefore, glycogen catabolism was greatly delayed in the ∆*Cfvps35* mutant.

### CfVps35 is involved in the cell wall stress response

**Fig. 6:**
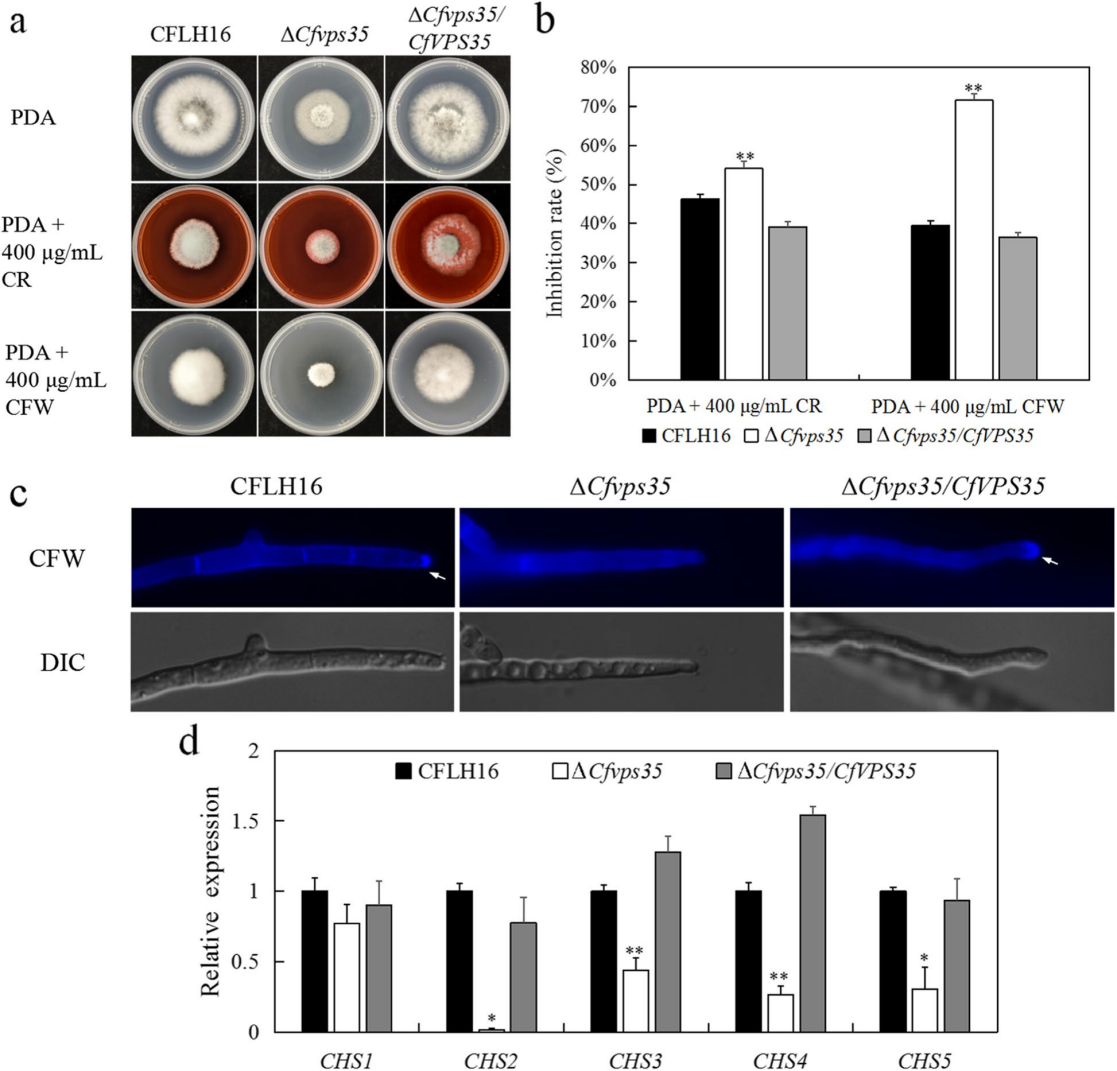
CfVps35 is involved in maintain of cell wall integrity. **a** The WT strain, the Δ*Cfvps35* mutant, and the complemented one were inoculated in PDA and PDA containing 400 μg/ml CFW or CR. **b** Statistical analysis of inhibition rates of the strains to cell wall stress. **c** The deposition of chitin in mycelia. (**d**) Relative expression levels of *CHS* genes in Δ*Cfvps35* mutant. Error bars are standard deviation and asterisks represent significance at *P*<0.01 (**)

During growth and development, the fungi are continually subjected to various environmental stresses. We further tested the sensitivity of strains under cell wall stress conditions. The inhibition rates of the mutant on calcofluor white (CFW) and congo red (CR) plates, were significantly higher (73 and 54%, respectively) than those of the WT and complemented strains (Fig. 6a and b). These results indicated that loss of the *CfVPS35* gene affected cell wall integrity.

Chitin is the main component of the cell wall of filamentous fungi. The distribution of chitin in the cell wall of mutants was analyzed by CFW staining. The results showed that the distribution of chitin on the mycelium tip was observed in the WT strain, but not in mutant (Fig. 6c). Further analysis of the genes for chitin synthesis in the ∆*Cfvps35* mutant by qRT-PCR showed that the expression of *CHS2*, *CHS3*, *CHS4* and *CHS5* genes was significantly down-regulated. Moreover, the expressions of the complemented strain restored to the level before deletion (Fig. 6d). These results revealed that *CfVPS35* is involved in maintain of cell wall integrity.

## Discussion

*C. fructicola* is the dominant pathogen causing anthracnose in *Ca. oleifera* [15]. In the past, the Vps35 protein was well studied in yeast and mammals and confirmed to play an important role in regulating various aspects of cell development [16–18]. However, the function of Vps35 in *C. fructicola* is unclear. In this study, we identified a CfVps35 protein homologous to the retromer subunit Vps35 of *S. cerevisiae.* Phylogenetic analysis and domain prediction revealed that Vps35 shows high conservation among fungi.

Vps35 protein is required for filamentous fungal growth. In *Magnaporthe oryzae*, the growth rate of *MoVPS35* gene deletion mutants decreased [19]. However, in *Fusarium graminearum*, the ∆*Fgvps35* mutants showed serious defects, such as slow growth rates and irregular shapes [20]. Similar to the growth phenotypes of these fungi, we found that the ∆*Cfvps35* mutant exhibited a significant decrease in the growth of the diameter of the mycelium, as well as that of aerial hyphae. These results show that Vps35 is highly conserved in filamentous fungi with different lifestyles. On the other hand, studies in *M. oryzae* indicate that the absence of any component of the cargo-recognition complex cause defects similar to those found in this study. Therefore, the role of Vps35 is probably the same in filamentous fungi, and it is as indispensable as Vps26 and Vps29.

Plants can recognize pathogen-associated molecular patterns (PAMPs) released by pathogens andactivate a series of defense responses, including the production of reactive oxygen species and diverse anti-microbial secondary metabolites [21, 22]. When the mycelium grows and adapts to the external environment, the cell wall needs to maintain a certain homeostasis, and previous studies have found that its integrity has profound implications for fungal pathogenicity [23]. In plant pathogenic fungi, the development of invasion structure, the growth and morphology of mycelia depend on the regular synthesis and distribution of chitin in the cell wall [24]. Both of CR and CFW can interfere with cell wall assembly by binding with chitin, disrupt the stability of cell wall and produce cell wall stress [25, 26]. The ∆*Cfvps35* mutant showed higher sensitivity to cell wall inhibitors (CFW and CR), and its growth was significantly inhibited compared with the growth of the WT and complemented strains. Consistent with our results, ∆*Movps35* mutant show increased sensitivity to cell wall stress [19], suggesting that the protein has the same function in both fungi. On the other hand, the absence of accumulation of chitin at the hyphal tip in the mutant, and the decreased genetic expression of chitin synthase, suggest that the retromer complex function it is necessary for correct gene expression of chitin synthase and therefore to cell wall integrity. Moreover, the critical role of CfVps35 in the cell wall integrity suggest the function of retromer complex in pathogenicity, the specific mechanism of this function requires further study.

In plant pathogenic fungi, the conidia are the main source of initial infection and sustained transmission. Therefore, the ability to produce conidia is vital to the prevalence of the disease. Previous studies showed that sporulation was significantly reduced in ∆*Movps35* and ∆*Fgvps35* mutants in *M. oryzae* and *F. graminearum* [19, 20]. In *C. fructicola*, the sporulation of the ∆*Cfvps35* mutant was significantly decreased compared with that of the WT and complemented strain, indicating that CfVps35 participates in the regulation of sporulation. These data indicated that vps35 might share the same regulatory models in different plant pathogenic fungi.

Appressorium is the key fungal structure to infect plant tissues [27]. The appressorium penetrates the cuticle and cell wall of plants. In *M. grisea*, the deletion of the *MGA1* gene related to the formation of the appressorium, resulted in a complete loss of pathogenicity of this fungus [28]. On the other hand, the ∆*Clpls1* mutants of *C. lindemuthianum* do not have defects in formation and maturation of the appressorium but may be deficient in the formation and/or positioning of the penetrating pore and therefore also lose pathogenicity [29]. In previous studies on *C. fructicola* we found that the *CfGCN5*, *CfVPS39*, and *CfVAM7* genes were involved in the regulation of appressorium development and the mutants completely lost their pathogenicity [1, 30, 31]. In this study, the appressorium formation rate of the ∆*Cfvps35* mutant was significantly reduced and its collapse rate was higher than that of the WT and complemented strain. Considering that CfVps35 is a component of the cargo-recognition complex, these results revealed that in *C. fructicola* the function of this complex is fundamental for the appressorium formation and the internal turgor pressure that this pathogen requires to invade its host plants.

Glycogen is one of the most abundant storage products in the conidia of *C. fructicola*. Glycogen is gradually transferred from conidia to appressorium and rapidly degraded [14]. The production of turgor requires the accumulation of a large amount of glycerol, which is derived from the transformation of energy substances such as glycogen, trehalose and lipid metabolism in conidia [14]. In *M. oryzae* it has been reported that glycogen transport and degradation are regulated by the *AGL1* and *GPH1* genes, which encode amyloglucosidase and glycogen phosphorylase respectively, and whose deletion inhibits glycogen metabolism and reduces virulence [32]. In this study, glycogen was gradually metabolized by the WT and complemented strain during appressorium development. In contrast, the Δ*Cfvps35* mutant showed a large amount of glycogen in conidia and appressorium, suggesting restriction of the glycogen metabolism. Therefore, because the appressorium turgidity of the ∆CfVps35 mutant is abnormal, its failure leads to loss of pathogenicity. In *M. oryzae*, the *MoVPS35* gene deletion mutants were able to cause weak lesions on wounded leaves, but almost no obvious lesions on unwounded leaves [19]. Similarly, in *F. graminearum*, the *FgVPS35* gene deletion also showed reduced virulence [20]. In contrast, in our study the ∆*CfVps35* mutant lost its cellophane penetrating ability and its pathogenicity in both wounded and unwounded leaves. It should be noted that loss penetrability is not the only possible cause of loss of virulence. Considering the loss of cell wall integrity in the ∆*Cfvps35* mutant, it can release PAMPs that can activate the immunity of the plant and thus the resistance [21]. It may also be the reason why the Δ*Cfvps35* mutant is unable to infect the wounded leaves. Therefore, we speculate that the loss of *CfVPS35* gene caused the defect of retromer complex, which indirectly led to loss of penetrability and pathogenicity of *C. fructicola.* In conclusion, previous studies and our current study have shown that vps35 plays key roles in development and pathogenicity in different fungal pathogens, suggesting that retromer complex is potentially effective means to control fungal diseases.

## Conclusion

In this research, a *S. cerevisiae VPS35* homologous gene *CfVPS35* was cloned from *C. fructicola*. It was found that the gene plays an important role in growth, sporulation, appressorium formation and stress responses, especially having defects in glycogen metabolism and turgor generation during appressorial development, further affecting pathogenicity of *C. fructicola.*

## Materials and Methods

### Strains and culture conditions

*Colletotrichum fructicola* CFLH16 was used as the WT (Wild Type) in this study for transformation. The WT strain, gene deletion mutant ∆*Cfvps35*, and complemented strain Δ*Cfvps35/CfVPS35* were maintained on PDA (Potato Dextrose Agar) at 28 ℃ for mycelial growth.

### Phylogenetic analysis and domain prediction

The sequences of the Vps35 proteins of *C. gloeosporioides*, *C. viniferum*, *C. siamense*, *C. higginsianum*, *Neurospora crassa*, *Fusarium proliferatum*, *Fusarium oxysporum*, *Verticillium nonalfalfae*, *Saccharomyces cerevisiae*, *Magnaporthe oryzae*, and *C. fructicola* were obtained from the NCBI database (https://www.ncbi.nlm.nih.gov/). The phylogenetic tree was constructed using the Mega 7.0 program [33]. The protein sequences obtained from NCBI were submitted to MEME Suite (https://meme-suite.org/) for motif prediction [34], and the Batch CD-search in NCBI was used to predict the conserved domain of these proteins. The analysis results were displayed using TBtools software [34].

### Targeted gene deletion and complementation

To generate the *CfVPS35* gene replacement construct, the *CfVPS35* DNA fragments of 1.0 kb upstream and downstream were amplified with primer pairs *CfVPS35-*1F/2R and *CfVPS35-*3F/4R (Table S1), respectively. The fragment of hygromycin (1.4 kb) was amplified with the primer pair Hyg F/R. The three fragments were joined using overlap PCR with the primer pair *CfVPS35*-1F/4R, purified and transformed into the protoplasts of WT by polyethylene glycol (PEG) mediated transformation [35]. The protoplast transformation method was based on a system in *F. graminearum* [36]. The PCR reaction designed in this study and the following conditions were used: 5 min at 95 ℃; 32 cycles of 95 ℃ for 30 s, 56 ℃ for 30 s, and 72 ℃ for 3 min; a final extension at 72 ℃ for 10 min. Cultured in TB_3_ medium (Yeast extract 3 g, Casamino acid 3 g, sucrose 200 g and 1.5% Agar) containing 4 μl/mL hygromycin for 2-3 days. Transformants with hygromycin resistance were screened by PCR using primer pairs *CfVPS35*-5F/H855R and *CfVPS35*-7F/8R (Table S1).

For the preparation of the complemented strain, the full length of *CfVPS35* and its native promoter region were amplified with the primer pair *CfVPS35*-9F/10R (Table S1). The PCR products were inserted in PYF11 vector (which includes the bleomycin resistance gene and the GFP green fluorescent gene) and were transformed into yeast-competent cells XK-125 [37]. The positive clones were identified by PCR using *CfVPS35*-7F/GFP-R primers (Table S1). The plasmids were transformed into *Escherichia coli* DH5α competent cells to generate the complementation vector. The correct sequenced vectors were transformed into protoplasts of the ∆*Cfvps35* mutant. The transformants that could grow on TB_3_ medium containing 2 μl/mL bleomycin were screened by green fluorescence under fluorescence microscope (ZEISS, Axio Observer) and verified by PCR to identify the complementary strains Δ*Cfvps35/CfVPS35* and determine the related phenotypes.

### Growth, sporulation, and appressorium formation

The WT strain CFLH16, gene deletion mutant ∆*Cfvps35*, and complemented strain Δ*Cfvps35/CfVPS35* were inoculated on PDA and MM [30] (7 mM NaNO_3_, 7 mM KCl, 617 μM MgSO_4_·7H_2_O, 11 mM KH_2_PO_4_, 29 μM Vitamin B1, 55 mM Glucose, 0.1% 1000 × trace elements and 1.5% Agar). The plates were kept in the incubator at 28 ℃ for 3 days. The diameters and heights of mycelial growth were measured.

For sporulation and appressorium formation, the strains were inoculated in PDB medium and shaken at 160 rpm for 48 h at 28 ℃. The conidia of each strain were collected and estimated using a blood cell counting board. The filtrate was centrifuged (Eppendorf 5415D) at 5000 rpm for 3 min, and the collected conidia were washed three times with ddH_2_O (Sterile double distilled water). The conidial suspensions (10^5^ spores/mL) were placed on hydrophobic coverslips at 28 ℃. The appressorium formation rate was determined 24 h later. The appressorium was treated with 20 μL of glycerin (2 mol/L), and the collapse rates were estimated after 10 min.

### Cell wall integrity assays

The strains were inoculated onto PDA with 400 μg/mL CR or CFW and cultured in the dark for 3 days at 28 ℃. The sensitivity was assessed by measuring the the diameter of the mycelial growth. The young mycelia were stained with 10 mg/mL CFW for 10 min without light, rinsed with ddH_2_O, and observed and photographed under a fluorescence microscope(Axio Observer 3; Carl Zeiss AG).

### Pathogenicity assays

The mycelial blocks or conidial suspensions (3×10^5^ spores/mL) of the strains were inoculated onto the detached leaves of *Ca. oleifera*. The inoculated leaves were kept under high-moisture conditions at 28 ℃ for 3-5 days. The lesions were observed and measured.

### Cellophane penetration assay

Circular disks of suitable size were cut from a cellophane sheet and placed on a PDA medium. The strains were inoculated to the center of cellophane and grown at 28 ℃ for 2 days. Then, the cellophane disks were taken out. The penetration of cellophane was observed after 2 days of culture at 28 ℃[1].

### Glycogen dyeing assay

The conidial suspensions (10^5^ spores/mL) were placed on hydrophobic coverslips to induce appressorium formation and dropped in the KI/I_2_ solution (1 mL of distilled water with 120 mg KI and 20 mg I_2_) for 24 h. Yellowish-brown glycogen deposits were observed in bright field. Quantitative analysis of staining using Image J[38].

### Real-time quantitative reverse transcription PCR analysis

Total RNA was extracted using RNAprep Pure Plant Kit (TIANGEN) including DNase treatment*.* And cDNA synthesis was carried out using the HiScript® 1st Strand cDNA Synthesis Kit. (Vazyme). Primers pair Actin F/R was used for amplification of the actin gene (Table S1). Real-time quantitative reverse transcription PCR (qRT-PCR) was performed to detect the expression level of the chitin synthase genes *CHSs* (Table S1). SYBR green–based quantitative PCR was performed using an Quant Studio 3 machine (Applied Biosystems). The comparative CT method was used for comparative quantitative comparison [39].

### Statistical analysis

All experiments were carried out at least three times, and each treatment had three replicates. All data were expressed as mean ± standard deviation (SD) and analyzed using SPSS 26.0. One-way ANOVA was used to determine differences among groups.

## Supplementary Information

**Additional file 1.**

## Data Availability

All data generated or analyzed during this study are included in this published article and its supplementary additional files. The complete amino acid sequence of CfVps35 analysed during the current study are available in the NCBI repository [Accession number: XP_031881347.1]. Other data generated or analyzed during this study are included in the article and its supplementary information files.

## Abbreviations

CR: Congo red
CFW: Calcofluor White
PCR: Polymerase chain reaction
PDA: Potato Dextrose Agar
PDB: Potato Dextrose Broth
MM: Minimal Medium
MM-N: Minimal Medium without NaNO_3_
