# Supplementary material for: Retromer subunit, CfVps35 is required for growth development and pathogenicity of Colletotrichum fructicola
Source: BMC Genom Data. 2022 Aug 28;23:68. doi: 10.1186/s12863-022-01084-4 (PMC9420259; doi:10.1186/s12863-022-01084-4)
Supplement: Supplementary file 1 — Additional file 1. [file 12863_2022_1084_MOESM1_ESM.docx]

**Supplementary information**

Table S1. Primers used in current study

| Primer | Sequence （5’-3’） | Purpose | Origin |
| --- | --- | --- | --- |
| *CfVPS35*-1F | TAGGTAGGCAGTAAGCGCAG | amplify *CfVPS35* 5’ flank sequence | designed in this study |
| *CfVPS35*-2R | TTGACCTCCACTAGCTCCAGCCAAGCC AACGGGAGTCGCAGACATGT |  |  |
| *CfVPS35*-3F | CAAAGGAATAGAGTAGATGCCGACCG GATGACCCGCCATCCATCTT | amplify *CfVPS35* 3’ flank sequence |  |
| *CfVPS35*-4R | AGCAATATCACCACGAACCT |  |  |
| *CfVPS35*-5F | AGAACGAACGAATCTGGCTG | validation of *CfVPS35* gene deletion |  |
| H855R | GCTGATCTGACCAGTTGC |  | [31] |
| *CfVPS35*-7F | GGCCCTTGTGAAGTTGCTTG | amplify *CfVPS35* gene sequence | designed in this study |
| *CfVPS35*-8R | CGAGTCGGGTATGATTGCGA |  |  |
| *CfVPS35*-9F | ACTCACTATAGGGCGAATTGGGTACTCAAATTGGTTGGCTGTTGAGTCTCATCAAG | amplify complemented sequence |  |
| *CfVPS35*-10R | CACCACCCCGGTGAACAGCTCCTCGCCCTTGCTCACTTTGGGGTAGAGAACCAGGC |  |  |
| Hyg-F | GGCTTGGCTGGAGCTAGTGGAGGTCAA | amplify *HPH* sequence | [31] |
| Hyg-R | CGGTCGGCATCTACTCTATTCCTTTG |  |  |
| GFP-R | GACACGCTGAACTTGTGGCCGTT | validation of complemented sequence |  |
| *CHS1*-F | TTTGCAGCCGTCTTCATTGC | qRT-PCR | designed in this study |
| *CHS1*-R | TTGCAGAAGGCGTAGACGTT |  |  |
| *CHS2*-F | TCCGCCCCTCTGATTCCTAA | qRT-PCR |  |
| *CHS2*-R | ACATGAAGGAAGCCGCGTAA |  |  |
| *CHS3*-F | CTGGCGACGTTGGAAGTAGT | qRT-PCR |  |
| *CHS3*-R | GAGGTGTAAGGATCCGAGCG |  |  |
| *CHS4*-F | GAACATCGAGATGGCGCAAC | qRT-PCR |  |
| *CHS4*-R | CTCGCCGGACTCAGGTATTC |  |  |
| *CHS5*-F | CCCACAAGATGACGGACCTC | qRT-PCR |  |
| *CHS5*-R | GCGTCGAGGTAGAACTTGGT |  |  |
| ACTIN-QF | CCCCATCTACGAGGGTTTCG | qRT-PCR |  |
| ACTIN-QR | CGTCAGGAAGCTCGTAGGAC |  |  |


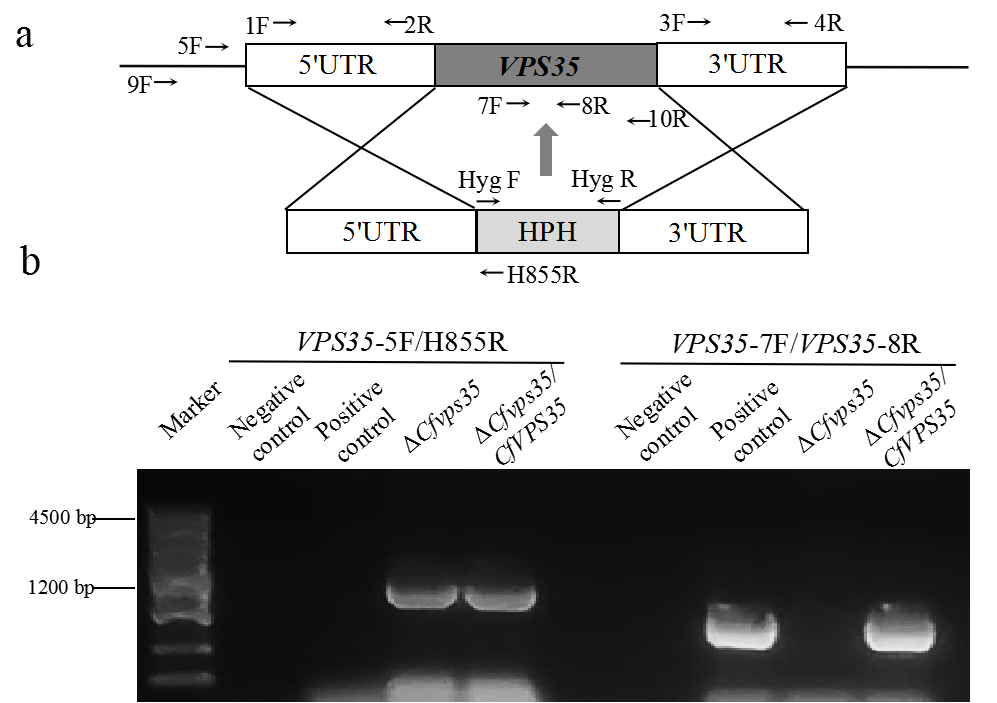


Figure S1 Targeted deletion of *CfVPS35* in *C. fructicola*.

1. Schematic illustration for deletion strategy of *CfVPS35* gene. (b) Validation of the gene deletion mutants by PCR amplified with primers 1 (*CfVPS35*-5F/H855R) and primers 2 ( *CfVPS35*-7F/8R) .
